# A Peculiar Experience

**Published:** 1897-06

**Authors:** 


### A PECULIAR EXPERIENCE.

UNDER the head of correspondence will be noted an important communication bearing upon a new aspect of the use of tuberculin as a diagnostic agent. While the fact of two cases arising at the same time, under identical circumstances, may seem more than a coincidence, it seems strange that no similar experiences have been recorded incidental to the testing of thousands of herds. Further, there has seemed no good reason at any time why there should be any precautionary advice given as to the use of the milk during the testing-period, for the injection of a sterilized product, free from germ-life, would make the same superfluous when considered in connection with the results all over the world, bearing testimony to the freedom from danger to mankind or the animals tested with tuberculin.

